# PReS-FINAL-2034: Α1 - Antitrypsin deficiency in children with arthritis: four cases

**DOI:** 10.1186/1546-0096-11-S2-P47

**Published:** 2013-12-05

**Authors:** C Pruunsild, J Ilisson

**Affiliations:** 1 Children's Clinic, Tartu University Hospital, Tartu, Estonia

## Introduction

α1 - antitrypsin (AAT) is a member of the serine protease inhibitor supergene family. The AAT deficiency is most often associated with the Z mutation and can contribute to the development of chronic obstructive pulmonary disease and liver pathology. The presence of arthritis with AAT deficiency has also been described.

## Objectives

To describe the cases of arthritis in children with AAT deficiency.

## Methods

The description of four cases. The AAT deficiency was found in three brothers (two with juvenile idiopathic arthritis (JIA), one reactive arthritis) and in a girl with JIA. While looking for possible cause(s) for hypertransaminasemia, the girl's and one of the brother's serum level of AAT was found to be below the normal range; the analysis of the other two brothers was ordered specially. In all cases the gene diagnostics was performed.

## Results

Arthritis in these children was diagnosed at the age of 8-15 years. All the three JIA patients have polyarticular course (two extended oligoarthritis, one subtype other arthritis), negative antinuclear antibodies, one has positive rheumatoid factor. No one had previous history of bronchitis. Two brothers (one with JIA) are overweight with body mass indexes as 26,3 kg/m^2 ^and 33,8 kg/m^2^. All the JIA patients have recieved disease modifying antirheumatic drugs (DMARDs): three plaquenil (2 in combination with trexane), one imuran. The serum levels of transaminases - ASAT and/or ALAT - were 1,5-2 times higher than the normal upper limit in two JIA patients. The other two patients had normal values. The girl with JIA had higher ASAT level just once before starting the DMARD, one brother presented with high values of ASAT and ALAT during the treatment. Three patients had AAT deficiency in repeated analyses (0.68-0.97 g/l). All the three brothers have the same mutation (in the protein E366K) in one gene copy of the AAT gene - they are carriers of the Z mutation (genotype Pi*MZ). The girl has S mutation (in the protein E288V) in one gene copy of the AAT gene - she is heterozygous for S mutation (genotype Pi*MS).

## Conclusion

Deficiency of AAT can be found in patients with arthritis. When performing blood tests seeking for cause(s) of hypertransaminasemia, the level of AAT should be checked; when the deficiency is present in repeated analyses, gene diagnostics should be done to specify the risk of organ damage and avoid the risk factors.

## Disclosure of interest

None declared.

